# Predicting patent challenges for small-molecule drugs: A cross-sectional study

**DOI:** 10.1371/journal.pmed.1004540

**Published:** 2025-02-12

**Authors:** Ally Memedovich, Brian Steele, Taylor Orr, Shanzeh Chaudhry, Mina Tadrous, Aaron S. Kesselheim, Aidan Hollis, Reed F. Beall

**Affiliations:** 1 Department of Community Health Sciences, Cumming School of Medicine, University of Calgary, Calgary, Alberta, Canada; 2 Leslie Dan Faculty of Pharmacy, University of Toronto, Toronto, Ontario, Canada; 3 Division of Pharmacoepidemiology and Pharmacoeconomics, Brigham and Women’s Hospital and Harvard Medical School, Boston, Massachusetts, United States of America; 4 Department of Economics, University of Calgary, Calgary, Alberta, Canada; London School of Economics and Political Science, UNITED KINGDOM OF GREAT BRITAIN AND NORTHERN IRELAND

## Abstract

**Background:**

The high cost of prescription drugs in the United States is maintained by brand-name manufacturers’ competition-free period made possible in part through patent protection, which generic competitors must challenge to enter the market early. Understanding the predictors of these challenges can inform policy development to encourage timely generic competition. Identifying categories of drugs systematically overlooked by challengers, such as those with low market size, highlights gaps where unchecked patent quality and high prices persist, and can help design policy interventions to help promote timely patient access to generic drugs including enhanced patent scrutiny or incentives for challenges. Our objective was to characterize and assess the extent to which market size and other drug characteristics can predict patent challenges for brand-name drugs.

**Methods and findings:**

This cross-sectional study included new patented small-molecule drugs approved by the FDA from 2007 to 2018. Market size, patent, and patent challenge data came from IQVIA MIDAS pharmaceutical quarterly sales data, the FDA’s Orange Book database, and the FDA’s Paragraph IV list. Predictive models were constructed using random forest and elastic net classification. The primary outcome was the occurrence of a patent challenge within the first year of eligibility. Of the 210 new small-molecule drugs included in the sample, 55% experienced initiation of patent challenge within the first year of eligibility. Market value was the most important predictor variable, with larger markets being more likely to be associated with patent challenges. Drugs in the anti-infective therapeutic class or those with fast-track approval were less likely to be challenged. The limitations of this work arise from the exclusion of variables that were not readily available publicly, will be the target of future research, or were deemed beyond the scope of this project.

**Conclusions:**

Generic competition does not occur with the same timeliness across all drug markets, which can leave granted patents of questionable merit in place and sustain high brand-name drug prices. Predictive models may help direct limited resources for post-grant patent validity review and adjust policy when generic competition is lacking.

## Abstract 2

Ally Memedovich and colleagues report on patent challenges within the first year of eligibility among small-molecule drugs approved by the FDA from 2007-2018 and investigate the extent to which market size and other drug characteristics predict such challenges.

## Author summary

**Why was this study done?:**

- The high cost of prescription drugs in the United States is sustained by brand-name manufacturers’ competition-free period, supported in part by patent protection, which generic drug competitors can challenge.
- Understanding the factors associated with such patent litigation can inform policies to promote timely generic competition.
- Categories of drugs overlooked by challengers, such as those with low market size, reveal policy gaps in which unchecked patent quality and high prices persist, warranting interventions like enhanced patent scrutiny or incentivizing challenges.

**What did the researchers do and find?:**

- Predictive models were constructed to determine the factors contributing to a patent challenge.
- Generic manufacturers’ drug patent challenges can be predicted with over 80% accuracy, with large market size being a strong positive predictor.
- Nearly half of all new drugs are unlikely to see a patent challenge from a generic competitor.

**What do these findings mean?:**

- The current process in the US for attracting timely generic competition to the pharmaceutical market does not work equally efficiently across all market segments, allowing low-quality patents to persist and maintaining high drug prices; particularly at risk are drugs with small market sizes.
- Limitations include the exclusion of patent characteristics and the exclusion of certain drug-related characteristics.

## Introduction

The United States has the largest market for brand-name pharmaceuticals internationally: It spends the most per capita on pharmaceuticals and brand-name prescription drug prices are over twice those of other OECD countries [1,2]. High pharmaceutical costs are sustained during the competition-free period guaranteed to manufacturers of new pharmaceuticals, allowing them to charge high prices to recoup development costs [3]. The main policy solution to reduce pharmaceutical spending in the US has been to encourage generic entry as soon as possible, through legislation and the US Food and Drug Administration (FDA) policies and guidance [4].

The competition-free period is defined by a combination of regulatory protections and patent protection [5]. For new patent-protected small-molecule drugs, the Hatch-Waxman Act prohibits the FDA from approving generic competitors until 4 years after the originator product’s FDA approval, although actual generic entry typically occurs much later [6,7]. Additionally, patents protect new drugs for 20 years from the application date, often starting before FDA approval [5,6]. To compensate for patent term loss during the regulatory approval process, the Hatch-Waxman Act allows one key drug patent to be extended by up to 5 years, resulting in a maximum competition-free period of up to 14 years post-FDA approval [8]. As a result, new brand-name drugs typically have a competition-free period of 12 to 14 years, although about one in 4 brand-name drugs can have exclusivity lasting over 17 years [9–12].

Generic entry typically occurs after patents expire, but it can happen sooner if patents are successfully challenged in court [7]. The Hatch-Waxman Act created the Abbreviated New Drug Application (ANDA) process for regulatory approval that allows generic companies to bypass clinical testing if they can prove that their products are bioequivalent to the corresponding brand-name drugs [13]. The FDA considers a “generic” to be a drug that contains the same active or key ingredient, same strength, uses the same dosage form, and uses the same route of administration as a brand-name drug [14]. Generic manufacturers must notify the FDA that they will not sell their product until after the brand-name patents expire or that the patents held on the brand-name drug are invalid or not infringed. This latter notification—a so-called Paragraph IV statement—is often challenged by the brand-name manufacturer, leading to litigation to determine whether the patents are enforceable [7]. If litigation commences, a 30-month stay of ANDA approval begins during which the FDA cannot approve a generic unless litigation is resolved or a settlement is reached [6,7]. The first generic company to successfully complete the Paragraph IV process is granted a 180-day market duopoly period with the originator, during which profitability remains high as prices experience only modest reductions with a single competitor [11,15]. If multiple companies submit ANDAs with Paragraph IV certifications on the same day, all successful challengers share the 180-day period before non-challenging manufacturers are allowed to enter the market [16].

Existing research examining the determinants of generic competition and Paragraph IV challenges show that market size is a critical factor in whether these challenges are brought [9–11,17–22]. While these studies help explain why the frequencies of challenges are not uniform across all drug types, they were not designed to predict the likelihood of drugs being targeted. Predictive modeling with cross-validation offers a stronger approach by testing how well the model performs on new data, unlike explanatory methods focused solely on past trends. FDA researchers recently used machine learning to predict the timing of ANDA applications, irrespective of patent challenges, and found the performance of the model was superior to traditional methods [18]; however, no study to our knowledge has focused these predictive methods on the Paragraph IV system and patent challenges specifically. This focus is important because patent challenges affect not only the FDA but also the courts, litigation costs, and both brand-name and generic pharmaceutical companies, which can make business decisions based on whether these challenges occur. It is also of clinical importance, as patent challenges affect how quickly patients have access to reasonably affordable medications. Further, identifying categories of drugs systematically overlooked by generic challengers, such as those with low market size, highlights gaps in which unchecked patent quality and high drug prices may persist, necessitating alternative policy interventions, such as enhanced patent scrutiny or incentivizing challenges. Therefore, our objective was to use a prediction model to characterize and assess the extent to which market size and other drug characteristics can predict patent challenges for brand-name drugs.

## Methods

### Design

In this cross-sectional study, we created a data set of patented small-molecule drugs approved in the US by the FDA from 2007 to 2018 and identified which drugs received a Paragraph IV patent challenge during the first year of eligibility (year 4 after market entry). Random forest and elastic net models were constructed to predict whether a drug patent was challenged within the first year of eligibility.

### Data sources and cohort construction

To construct our study cohort, we updated a previously published data set of small-molecule drugs approved between 2000 and 2016 [23], by incorporating drugs approved in 2017 to 2018. FDA reports [24] were used to identify drugs qualifying for special regulatory review programs (i.e., accelerated approval, breakthrough therapy, fast-track, priority review, and rare [Orphan Drug Act] disease designation) to provide potential indicators of clinical importance. Patent data were retrieved annually each January from 2000 to 2024 from the electronic archives of the FDA’s Approved Drug Products with Therapeutic Equivalence Evaluations (“Orange Book”). The initial data set was acquired in 2017 through a Freedom of Information Act request and has been maintained and updated annually with the most recent editions [25].

This article is based in part on internal analysis by the authors using IQVIA MIDAS quarterly sales data, which were obtained under license from IQVIA and reflect estimates of marketplace activity (Copyright IQVIA, all rights reserved). The statements, findings, conclusions, views, and opinions contained and expressed herein are not necessarily those of IQVIA. Annual market size estimates were drawn from the IQVIA MIDAS quarterly pharmaceutical sales value data for the United States for the period 2011 to 2022. The IQVIA MIDAS data is an IQVIA proprietary information service which integrates IQVIA’s national audits into a globally consistent view of the pharmaceutical market and provides estimated product volumes of registered medicines, trends, and market share through retail and non-retail channels. This market research information reflects local industry standard source of pack prices, which is average invoice price for the US; these prices do not take into account rebates or claw backs, details of which are normally confidential, and therefore, these estimated prices do not reflect net prices realized by the manufacturers. Sales values reflected in these IQVIA audits are calculated by applying such relevant pricing to the product volume data collected for, and reflected in, such audits. Market size in the year preceding challenge eligibility was adjusted for inflation to that of the last observation year (2022) using the corresponding CPI inflation metric [26]. Market values were not normally distributed; to account for this, we evaluated multiple functional forms (e.g., natural logarithms, quantiles, manually specified cut-points) and found that deciles performed best during model development. The IQVIA MIDAS data also provided therapeutic class data based on the WHO’s Anatomical Therapeutic Chemical (ATC) Classification System (level 1).

Based on prior research [27] showing that the majority of Paragraph IV challenges are filed within the first year that a drug becomes eligible and that our preliminary analysis of the current data set reflected the same phenomenon (**S1 Fig**), we focused on small-molecule drugs’ first year of patent challenge eligibility (i.e., year 4 following FDA approval) to simplify the analysis and improve interpretability. Given this decision and market data availability, only drugs approved between 2007 and 2018 were eligible for inclusion [24].

Route of administration data and patent data were retrieved from archives of the FDA’s Orange Book to determine drugs’ patent status during all years 2011 to 2022 [28]. Routes of administration were categorized as oral, injectable, or other (which contained products available in multiple forms [*n* = 14] and with less common routes [*n* = 20] like transdermal). Counts of non-duplicate patent numbers were calculated for each product and the date of its last-expiring patent was used for assessing challenge eligibility. To address the skewed distribution, the number of patents were categorized into quartiles using cut-points of 4, 6, and 11 patents.

For consistency with the previously published data set [22], only novel drugs (i.e., New Molecular Entities) were included, grouped into product portfolios by their stem tradename (e.g., the Abilify portfolio would also have subsequent versions Abilify Maintena Kit and Abilify Mycite Kit). We confirmed all included drugs had active prescription status and unexpired patents during the full year of observation in which an early challenge was possible.

The FDA’s Paragraph IV patent challenges list was used to determine challenge dates, serving as our outcome variable [29].

### Prediction models—Random forest and elastic net classification

Predictor variables included drugs’ market size, patent count, therapeutic class, route, and special regulatory designations. All analyses were conducted in R version 4.3.2. Models were compared using holdout validation (referred to as a test-train split), where models were developed and tuned on a training data set (a random sample of 80% of the data) and predictions were made with a test data set (the remaining 20%) to evaluate model performance. Model performance was reported for the Brier score (squared loss, a strictly proper scoring rule serving as the primary model performance metric), sensitivity, specificity, positive predictive value (PPV), negative predictive value (NPV), area under the curve (AUC), and misclassification error.

Random forest was constructed using the {ranger} package [30]. Using the training subset, the number of trees was tuned by iteratively increasing the number in the thousands (e.g., starting with 5,000 and adding an additional 1,000 trees) and then comparing estimates until out-of-bag error was consistent. We also evaluated the minimum number of variables to randomly sample at each node split (argument “mtry” from 3, 4, and 5); 3 had the best performance. Following tuning, the holdout random forest was used to evaluate performance. Using *ranger*’s built-in “holdout” argument with case weights (0 = test, 1 = train) [30], the reported out-of-bag error is equivalent to misclassification error (classification random forest) and Brier score (probability random forest). The {pROC} package [31] was used to estimate AUC. Finally, the permutation variable importance (PVI) was plotted for the random forest model [32].

As random forests are “black box” machine learning models, we developed a comparative, more readily interpretable elastic net binary classification model. Elastic net regularization was selected due to the smaller size of the data set, the availability of many predictor variables, and to account for possible collinearity among some of the variables (e.g., products’ market sizes and counts of associated patents, **S2 Fig**). Regularization is an alternative to using forward or backward selection to avoid overfitting and improve prediction. Using the training data, we evaluated a range of mixing penalties (the hyperparameter “mixing” the regularization penalties) between 0 (a ridge penalty) and 1 (a LASSO [Least Absolute Shrinkage and Selection Operator] penalty) and selected the parameter that minimized squared loss (e.g., the Brier score) [33,34]. Performance metrics were also calculated for ridge and LASSO models. The cross-validated elastic net model was then used to calculate the log likelihood of patent challenges based on the training data for all possible combinations of drug characteristics (made available via a data dashboard using the {shiny} packages in R [35]). Finally, the elastic-net model’s coefficients were estimated and are reported using the entire data set. Analyses were conducted using {glmnet} [36,37].

All code used for this project is available at: https://github.com/reedbeall/us-patent-challenge.

## Results

**Fig 1:**
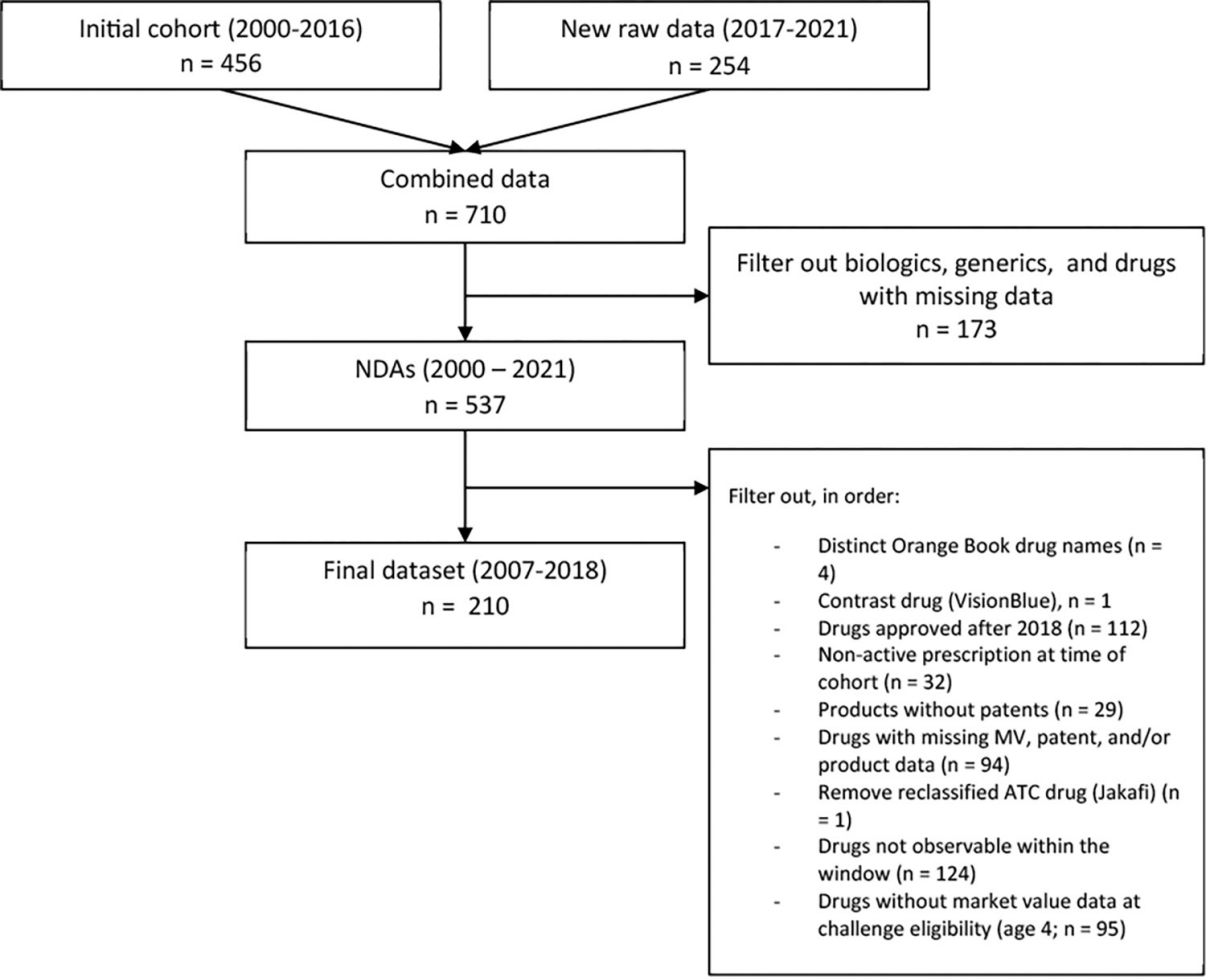
Flowchart of included drugs.

After excluding 41 drugs because market data were not available during the period of interest, our final cohort covered 210 new small-molecule, FDA-approved drugs from 2007 to 2018 (**Fig 1**). The median annual market size of the overall cohort was $111.3 million (IQR: $28.4 to $311.7 million) (**Table 1**). The median count of Orange Book-listed patents per drug was 6 (IQR: 4–10). The drugs covered 14 of the WHO’s main ATC classes, with the most common being antineoplastic and immunomodulating agents (*n* = 59, 28%), drugs for nervous system disorders (*n* = 29, 14%), and anti-infectives for systemic use (*n* = 27, 13%). The most common route of administration was oral (*n* = 141, 67%). About one-third were first-in-class (*n* = 59, 28%) and a similar number had a rare disease drug designation (*n* = 66, 31%). The drugs covered all FDA special review programs, including accelerated approval (*n* = 25, 12%), breakthrough therapy (*n* = 28, 13%), fast track (*n* = 66, 31%), and priority review (*n* = 101, 48%). Among the 210 drugs, 57% (*n* = 119) experienced a Paragraph IV patent challenge within the first year of eligibility.

**Table 1:** Descriptive statistics by challenge status (observation window of 2011–2022).

Characteristic	Challenged, *n* = 119	Not challenged, *n* = 91	Challenge percentages
**Year 4 market value in millions (median, IQR)**	$202.1 ($77.3–$562.2)	$40.5 ($13.8–$138.8)	57%
**Market value deciles**			
1 ($9,247–$7,734,174)	5	16	24%
2 ($7,734,175–$21,572,630	5	16	24%
3 ($21,572,631–$38,422,756)	8	13	38%
4 ($38,422,756–$75,481,061)	11	10	52%
5 ($75,481,062–$111,326,185)	12	9	57%
6 ($111,326,186–$160,633,818)	13	8	62%
7 ($160,633,819–$234,890,241)	16	5	76%
8 ($234,890,242–$483,920,217)	15	6	71%
9 ($483,920,218–$1,002,587,866)	19	2	90%
10 ($1,002,587,866–$9,471,629,567)	15	6	71%
**Number of patents**			
1–3	28 (22%)	20 (22%)	58%
4–5	22 (17%)	24 (26%)	48%
6–10	42 (32%)	25 (27%)	63%
11+	27 (21%)	22 (24%)	55%
**WHO ATC class**			
(A) Alimentary tract and metabolism	17 (14%)	8 (9%)	68%
(B) Blood and blood forming organs	9 (8%)	5 (5%)	64%
(C) Cardiovascular system	9 (8%)	2 (2%)	81%
(G) Genitourinary and hormones	5 (4%)	7 (8%)	42%
(J) Anti-infectives	4 (3%)	23 (25%)	15%
(L) Antineoplastic and immunomodulators	34 (29%)	25 (27%)	57%
(N) Nervous system	24 (20%)	5 (5%)	83%
Other	17 (14%)	16 (18%)	52%
**Route of administration**			
Injectables	17 (14%)	21 (23%)	45%
Oral	88 (74%)	53 (59%)	62%
Other	14 (12%)	17 (19%)	45%
**Drug and regulatory characteristics**			
First-in-class drug	35 (29%)	24 (26%)	59%
Accelerated approval	13 (11%)	12 (13%)	52%
Priority review	49 (41%)	52 (57%)	46%
Fast-track	25 (21%)	41 (45%)	38%
Breakthrough therapy	13 (10%)	15 (15%)	46%
Orphan Drug Act designation	36 (30%)	30 (33%)	55%

Market size estimates and therapeutic class data are based on IQVIA MIDAS quarterly volume sales data for the US, reflecting estimates of marketplace activity. Copyright IQVIA. All rights reserved.

### Predictive model performance

The random forest prediction model predicted 81% of test cases correctly, outperforming the elastic net model on almost every calculated metric, with a Brier score of 0.178, an AUC of 0.807, and a misclassification error of 0.190 (**Tables 2 and S1**). The best-performing model using the elastic net approach had a mixing penalty of 0.95. The elastic net model predicted 76% of test cases correctly, had a Brier score of 0.369, and an AUC of 0.770. Elastic net had higher sensitivity (0.926) than random forest (0.815) but much lower specificity (elastic net: 0.400, random forest: 0.706). Elastic net performance was comparable to LASSO and was better than ridge regularization (**S2 Table**).

**Table 2:** Model classification performance.

Performance metric	Random forest	Elastic net
**Brier (squared loss)**	0.177	0.369
**Sensitivity**	0.815	0.926
**Specificity**	0.706	0.400
**PPV**	0.880	0.735
**NPV**	0.800	0.750
**AUC**	0.807	0.770
**Misclassification**	0.190	0.238

AUC, area under the curve; NPV, negative predictive value; PPV, positive predictive value.

### Results of the predictive models

**Fig 2:**
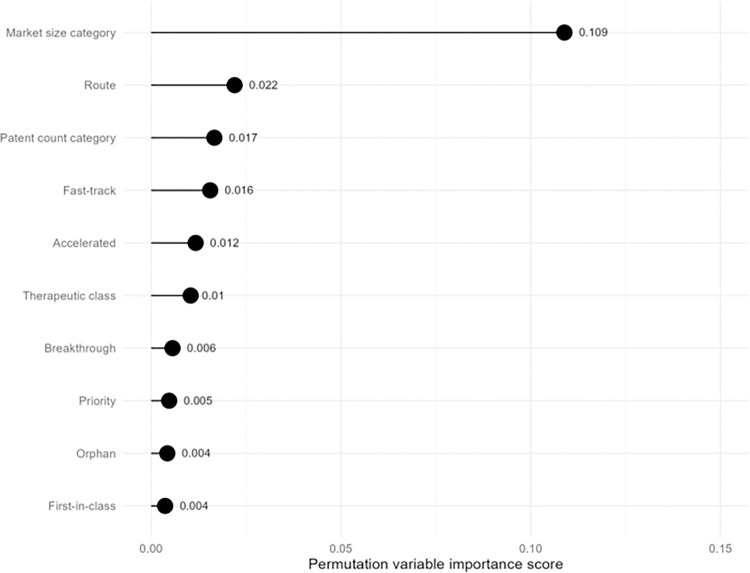
Variable importance analysis using random forest classification.

Market size was most important for classification (PVI: 0.109) (**Fig 2**). The random forest-estimated variable importance metric (PVI) for market size was approximately 5 times larger than the second-largest value, route (PVI: 0.022), and approximately 6 times larger than the third-largest value, patent count (PVI: 0.017). PVI were largely comparable between classification and probability forests (**S3 Table**).

Similar results were observed in the elastic net model. Important positive predictors were market size deciles (decile 9 coefficient: 1.26; decile 10 coefficient: 0.58) and the ATC class for drugs that affect the cardiovascular system (coefficient: 0.64) and nervous system (coefficient: 0.41) (**Table 3**). Important negative predictors (log likelihoods associated with drugs not receiving patent challenges) for the final model were the ATC class for anti-infectives (coefficient: −1.57), the lowest deciles for market value (decile 1 coefficient: −1.16; decile 2 coefficient: −1.24), and the fast-track approval designation (coefficient: −0.65) (**Table 3**).

The predictions for both models are available for 61,440 scenarios based on unique permutations of the drug characteristics considered in the analysis (Online Drug Patent Challenge Prediction Abacus). The Random Forest predicted a patent challenge as the most likely outcome in 49% (30,376) of the contingencies considered and the Elastic Net predicted a challenge in 57% (34,992).

**Table 3:** Elastic net predictive model coefficients and test classifications.

	Random forest				Elastic net (intercept: 0.77)			
Characteristic	Correctly classified	False positive	False negative	Coefficient		Correctly classified	False positive	False negative
**Annual market value at challenge eligibility (deciles)**								
1 ($9,247–$7,734,174)	2	1	1	−1.16		2	2	
2 ($7,734,175–$21,572,630	6			−1.24		3	3	
3 ($21,572,631–$38,422,756)	3		1	−0.66		3	1	
4 ($38,422,756–$75,481,061)	2			−0.12		2		
5 ($75,481,062–$111,326,185)	4			.		3		1
6 ($111,326,186–$160,633,818)	2	1		.		1	2	
7 ($160,633,819–$234,890,241)	5			0.28		4	1	
8 ($234,890,242–$483,920,217)	3		2	.		5		
9 ($483,920,218–$1,002,587,866)	2		1	1.26		3		
10 ($1,002,587,866–$9,471,629,567)	5	1		0.58		5		1
**Number of patents**								
1–3	8	1	1	0.05		7	3	
4–5	9		1	.		6	3	1
6–10	12	1	1	−0.01		12	2	
11+	5	1	2	.		6	1	1
**WHO ATC class**								
(A) Alimentary tract and metabolism	4		1	0.17		4	1	
(B) Blood and blood forming organs	1	1		.		2		
(C) Cardiovascular system	4			0.64		3	1	
(G) Genitourinary and hormones	2		1	−0.17		2	1	
(J) Anti-infectives	5			−1.57		3		2
(L) Antineoplastic and immunomodulators	9		1	.		8	2	
(N) Nervous system	5	1	1	0.41		6	1	
Other	4	1	1	−0.08		3	3	
**Route of administration**								
Injectables	11			.		8	2	1
Oral	19	2	4	.		20	4	1
Other	4	1	1	−0.19		3	3	
**Drug and regulatory characteristics** *								
First-in-class drug	12	1		.		10	3	
Accelerated approval	5		1	.		5	1	
Priority review	17		2	.		16	2	1
Fast-track	10		1	−0.65		10	1	
Breakthrough therapy	1		1	−0.24		2		
Orphan Drug Act designation	10		2	.		10	2	

*Categories are neither exclusive nor required.

The magnitude and positive/negative sign are indicative of the variable’s relationship with the outcome. Results should be interpreted with caution given the small test data set and imbalanced classes. Annual market deciles and therapeutic class data are based on IQVIA MIDAS quarterly volume sales data for the US, reflecting estimates of marketplace activity. Copyright IQVIA. All rights reserved.

## Discussion

We developed Two models to predict Paragraph IV patent challenges in the first year of eligibility using supervised machine learning techniques and found these could be predicted with between 81% (random forest) and 76% (elastic net) accuracy. Market size was a main driver for prediction across both prediction models. From the elastic net model, variables associated with lower predictions of receiving challenges also included whether drugs came from the anti-infective ATC class or qualified for the FDA’s fast-track program that expedites drug development.

Our study is the first to our knowledge to investigate whether FDA-expedited approval programs can predict patent target potential for generics. We found fast-track and other drugs in special review programs attract significantly fewer generic competitors. Prior research has demonstrated that “Program Specific Guidance” published by the FDA for bioequivalence applications is associated with early generic interest [17,18]. But as special review drugs can be subject to ongoing manufacturer or FDA monitoring or testing for adverse effects, the FDA may be less likely to issue such guidance during their early years on the market compared to drugs without special guidance.

Our finding that market size is a predictor of patent challenges corroborates prior research [9–11,19,20]. Existing studies have focused on identifying drugs to receive patent challenges; however, it is also important for policymakers to address drugs without challenges. Our study found 43% of all new patented drugs included in the sample were not subject to patent challenges in the first year of eligibility. Furthermore, there are a substantial number of future scenarios in which our models suggest that a patent challenge is not the most likely outcome (51% or 43% according to the Random Forest or Elastic Net models, respectively). Challenging patents is resource-intensive, with the potential benefits needing to justify the costs. Litigation costs are not linear and depend on the amount of money at risk: for less than $1 million at risk, the median total pre- and post-trial legal costs are $900,000; but for more than $25 million at risk, these costs only rise to $5 million [38]. Targeting larger markets is rational, especially given that the generic business model assumes mass production. Because all patents listed in the Orange Book must undergo Paragraph IV processes to attain early market entry and filing a Paragraph IV certification constitutes infringement, any patent poses important barriers, even if it is later found invalid or uninfringed.

The clinical implication of this work is that patients often wait longer to get access to cost-lowering generic competition, which can affect clinical outcomes. In the US, up to 35% of adults report cost-related nonadherence to prescription medication, leading to a 15% to 22% increase in all-cause mortality [39–42]. The cost of prescription drugs continues to be an important policy issue; for example, from 2022 to 2023, more than 4,000 drugs had price increases, 46% of which were larger than the rate of inflation, while only about 1,600 had price decreases [43]. The average price increase was 15.2%, translating to almost $600 per drug [43]. Ensuring patients have timely access to affordable medications is paramount to patient health. Our analysis showed that drugs with smaller markets are less likely to receive a patent challenge, and therefore, may be less likely to receive timely generic entry. These markets may represent markets in which patients already have limited options, and the traditional method of waiting for generic entry to occur may not be enough to ensure patients have access to necessary treatments.

A primary policy implication of our work is that generic competition cannot be solely relied upon to test the quality of drug patents held by brand-name manufacturers and subsequently reduce excessive drug prices. When patent challenges are infrequent (i.e., for small markets), other tools must be used. In 2017, Congress acknowledged the importance of generic entry to reduce drug prices and recognized that some drugs will not attract a high level of generic competition by introducing the competitive generic therapy (CGT) pathway [16]. A generic firm may request a CGT-designation for a drug with “inadequate generic competition,” defined as a drug for which there is only 1 approved drug included in the Orange Book [16]. If granted, the FDA may expedite the development and review of an ANDA. Further, CGT-designated drugs for which there are no unexpired patents at the time of ANDA submission may receive a 180-day early market entry period [16]. However, this process, while incentivizing generic entry into potentially undesirable markets, only applies to drugs for which there is no unexpired patents or exclusivities listed in the Orange Book [16]. Therefore, generic firms must still wait for patents to expire to enter the market, potentially leaving patients waiting years to receive adequately priced drugs.

One strategy to incentivize Paragraph IV patent challenges could be to increase the 180-day period for generic manufacturers to challenge patents in smaller markets. Under the Paragraph IV structure, the first generic to successfully challenge a patent and reach the market receives a 180-day early market entry period with respect to non-patent challenging generics, but this timeframe could be lengthened for smaller markets with a low probability of seeing Paragraph IV certifications. For markets unlikely to receive timely competition, other policy tools, such as price negotiation, could restrain pricing. One proposed strategy involves designing flexible regulatory protection periods. In this approach, the regulatory authority would guarantee an exclusive market to manufacturers in exchange for lower prices—and the lower the price, the longer the protection period [44]. This can also be practiced de facto by payers (via contract) that can use predictive models like ours to identify which drugs are so unlikely to draw generics that other strategies should be pursued.

Our study had limitations. Many stem from the exclusion of variables that were not readily available publicly, will be the target of future research, or were deemed beyond the scope of this project, and we hope others will build on this work to explore additional data points and improve the model. For example, we focused on drug characteristics, rather than characteristics of the challenge cases (e.g., outcomes) or the associated patents, such as their type (i.e., compound, formulation, process, use), quality, or listing time [19,20]. Additionally, we only indirectly considered molecule and manufacturing complexity. Studies by FDA researchers reported using internal resources to rate complexity and found significant negative associations with generic entry [17,18]. We did not pursue measures of market share or other proxies for competitiveness, which may indicate where patent disputes are more likely. Furthermore, forecasting the number of challengers was not targeted, though we noted in **S2 Fig** that with larger market size comes more challengers and vice versa. As also underscored by FDA researchers [17], predicting the number of generic challengers is useful for anticipating submission volumes for resource allocation. A possible implication of this finding for further study is that challengers in smaller markets are more likely to be sole challengers, which may lead to a more lucrative 180-day duopoly period. Our study did not consider any changes in FDA policy or market dynamics or any new legislation that may have affected the nature and quantity of ANDA submissions and Paragraph IV statements during the study period. Our study does not assess the extent to which Paragraph IV challenges accelerate generic entry or affect other market dynamics, questions we plan to explore in future research. Our study focused on the most well-known avenue for challenging market protection, though other pathways exist, such as “section VIII” statements enabling earlier generic entry for specific indications (i.e., “skinny labels”) [45] and “paper NDAs” (available via the 505(b)(2) pathway), which allow market entry with reduced clinical testing for drugs similar but not identical to existing approved products [46,47]. Finally, model performance should be interpreted with caution as excellent performance metrics from a single sample may not transfer to new settings [48]. Future work should explore how these models perform with drug patent challenges, and the impact of those challenges, in different jurisdictions and other years.

## Conclusion

Patients and insurers rely on generics to reduce the price of expensive medications. Predictive models, based on machine learning and which incorporate market size and other characteristics, can aid in directing resources for patent review and anticipating generic competition. These findings suggest the need for different policy tools to manage drug costs in market segments more or less likely to attract generic interest.

## Supporting information

S1 Fig Density plot of the proportion of 914 challenges observed by years after FDA approval. (TIF)

S2 Fig Correlation matrix heatmap. (TIF)

S1 Table Comparison of model performance for random forest, elastic net, LASSO, and ridge models. (DOCX)

S2 Table Predictive model confusion matrices. (DOCX)

S3 Table Permutation variable importances for random forests. (DOCX)

## Abbreviations

ANDA: Abbreviated New Drug Application
ATC: Anatomical Therapeutic Chemical
AUC: area under the curve
CGT: competitive generic therapy
FDA: Food and Drug Administration
LASSO: Least Absolute Shrinkage and Selection Operator
NPV: negative predictive value
PPV: positive predictive value
PVI: permutation variable importance
