# Supplementary figures and images for: Predicting patent challenges for small-molecule drugs: A cross-sectional study
Source: PLoS Med. 2025 Feb 12;22(2):e1004540. doi: 10.1371/journal.pmed.1004540 (PMC11867330; doi:10.1371/journal.pmed.1004540)

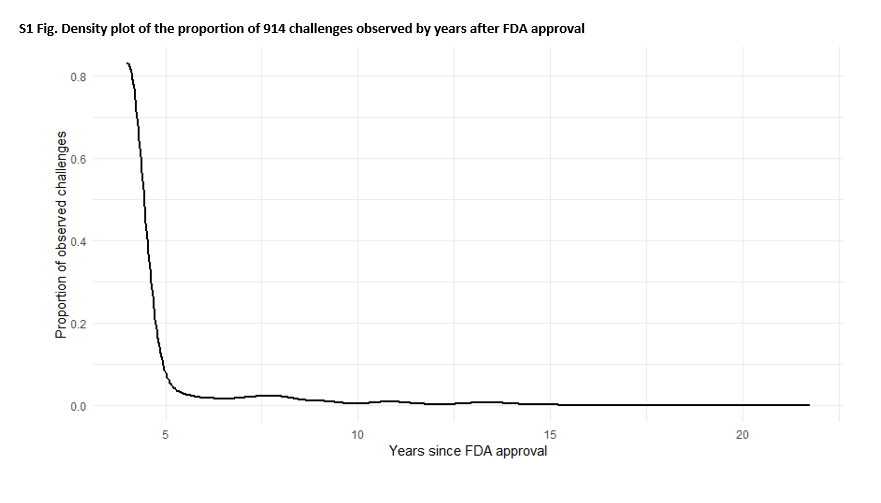

Supplement: S1 Fig — (TIF) [file pmed.1004540.s001.tif]

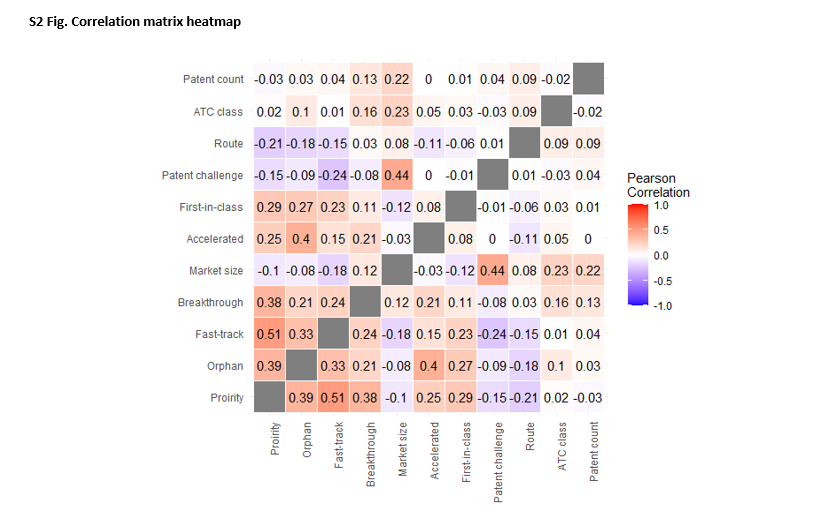

Supplement: S2 Fig — (TIF) [file pmed.1004540.s002.tif]
