# Supplementary material for: Predicting patent challenges for small-molecule drugs: A cross-sectional study
Source: PLoS Med. 2025 Feb 12;22(2):e1004540. doi: 10.1371/journal.pmed.1004540 (PMC11867330; doi:10.1371/journal.pmed.1004540)
Supplement: S1 Table — (DOCX) [file pmed.1004540.s003.docx]

**S1 Table. Comparison of model performance for random forest, elastic net, LASSO, and ridge models.**

| **Indicator** | **Brier**  **score** | **Sens.** | **Spec.** | **PPV** | **NPV** | **AUC** | **Misclass.** |
| --- | --- | --- | --- | --- | --- | --- | --- |
| **Random forest** | 0.178 | 0.815 | 0.706 | 0.880 | 0.800 | 0.807 | 0.190 |
| **Elastic net (alpha = 0.95)** | 0.369 | 0.926 | 0.40 | 0.735 | 0.750 | 0.770 | 0.238 |
| **LASSO (alpha = 1)** | 0.369 | 0.926 | 0.400 | 0.735 | 0.750 | 0.770 | 0.238 |
| **Ridge (alpha = 0)** | 0.380 | 0.815 | 0.600 | 0.786 | 0.643 | 0.753 | 0.333 |

Alpha refers to the elastic net mixing parameter. AUC: Area under the curve, Misclass.: Misclassification, NPV: Negative predictive value, PPV: Positive predictive value, Sens.: Sensitivity, Spec.: Specificity
