# Supplementary material for: Predicting patent challenges for small-molecule drugs: A cross-sectional study
Source: PLoS Med. 2025 Feb 12;22(2):e1004540. doi: 10.1371/journal.pmed.1004540 (PMC11867330; doi:10.1371/journal.pmed.1004540)
Supplement: S2 Table — (DOCX) [file pmed.1004540.s004.docx]

**S2 Table. Predictive model confusion matrices**

| **Random forest** | | | | |
| --- | --- | --- | --- | --- |
|  | | **True** | | **Total,**  **n (%)** |
|  |  | **Challenged,**  **n (%)** | **Not challenged,**  **n (%)** |  |
| **Predicted** | **Challenged** | 22 (52.4%) | 5 (11.9%) | 27 (61.9%) |
|  | **Not challenged** | 3 (0.1%) | 12 (28.6%) | 15 (35.7%) |
|  | **Total** | 25 (59.5%) | 17 (40.5%) | 42 (100%) |

| **Elastic net (**λ**=0.95)** | | | | |
| --- | --- | --- | --- | --- |
|  | | **True** | | **Total,**  **n (%)** |
|  |  | **Challenged,**  **n (%)** | **Not challenged,**  **n (%)** |  |
| **Predicted** | **Challenged** | 25 (59.5%) | 9 (21.4%) | 34 (81.0%) |
|  | **Not challenged** | 2 (0.05%) | 6 (14.3%) | 8 (19.0%) |
|  | **Total** | 27 (61.9%) | 15 (35.7%) | 42 (100%) |

Predictive models were trained on the random sample of 168 drugs and then assessed against the test data subset (n=42). These confusion matrices show the performance of trained models on test data.
