# Supplementary material for: Predicting patent challenges for small-molecule drugs: A cross-sectional study
Source: PLoS Med. 2025 Feb 12;22(2):e1004540. doi: 10.1371/journal.pmed.1004540 (PMC11867330; doi:10.1371/journal.pmed.1004540)
Supplement: S3 Table — (DOCX) [file pmed.1004540.s005.docx]

**S3 Table. Permutation variable importances for random forests**

| **Variable** | **PVI (classification forest)** | **PVI (probability forest)** |
| --- | --- | --- |
| Market value deciles | 0.109 | 0.084 |
| Route of administration | 0.022 | 0.011 |
| Patent count category | 0.017 | 0.007 |
| Fast track | 0.016 | 0.011 |
| Accelerated | 0.012 | 0.006 |
| ATC | 0.010 | 0.006 |
| Breakthrough | 0.006 | 0.002 |
| Priority | 0.005 | 0.001 |
| Orphan Drug Act | 0.004 | 0.002 |
| First in class | 0.004 | 0.001 |

Classification and probability random forests with identical hyperparameters were constructed to assess model performance (Brier score for probability forest)
